# Functional mass spectrometry indicates anti-protease and complement activity increase with COVID-19 severity

**DOI:** 10.3389/ebm.2025.10308

**Published:** 2025-01-29

**Authors:** Douglas D. Fraser, Swapan Roy, Matt Kuruc, Maritza Quintero, Logan R. Van Nynatten, Gediminas Cepinskas, Haiyan Zheng, Amenah Soherwardy, Devjit Roy

**Affiliations:** ^1^ London Health Sciences Centre Research Institute, London, ON, Canada; ^2^ Pediatrics, Western University, London, ON, Canada; ^3^ Biotech Support Group LLC, Monmouth Junction, NJ, United States; ^4^ Medicine, Western University, London, ON, Canada; ^5^ Medical Biophysics, Western University, London, ON, Canada; ^6^ Rutgers Center for Integrative Proteomics, Rutgers University, Piscataway, NJ, United States; ^7^ Nathan Littauer Hospital, Gloversville, NY, United States

**Keywords:** COVID-19, functional mass spectrometry, neutrophil elastase, lymphocyte granzyme B, complement

## Abstract

Investigations on some innate immunity proteins can yield misleading information, as investigators often rely on static measurements and assume a direct correlation to function. As protein function is often not directly proportional to protein abundance, and mechanistic pathways are interconnected and under constant feedback regulatory control, functional analysis is required. In this study, we used functional mass spectrometry to measure anti-protease and complement activity in plasma obtained from coronavirus disease 2019 (COVID-19) patients. Our data suggests that within 48 h of hospital admission, COVID-19 patients undergo a protease storm with significantly elevated neutrophil elastase (p < 0.001) and lymphocyte granzyme B (p < 0.01), while, anti-protease activity is significantly increased, including alpha-1 antitrypsin (AAT; p < 0.001) and alpha-1-antichymotrypsin (ACT; p < 0.001). Concurrently, the ratio of C3a to C3beta activity significantly decreased with increasing COVID-19 severity, suggesting more complement activation (Mild COVID-19 p < 0.05; Severe COVID-19 p < 0.001). Activity levels of AAT, ACT and C3a/C3beta remained unchanged over 10 hospital days. Our data suggests that COVID-19 is associated with both a protease storm and complement activation, with the former somewhat balanced with increased anti-protease activity. Evaluation of the AAT/ACT ratio and C3a/C3beta ratio indicated that COVID-19 severity is associated with both neutrophil elastase neutralization and complement activation.

## Impact statement

Our work is important as many investigations on innate immunity proteins can yield misleading information, as investigators often rely on static measurements. We used a novel approach which relies on functional mass spectrometry to measure the active site, thereby inferring function. We show that with increasing COVID-19 severity the anti-protease activity is significantly increased and that the ratio of C3a to C3beta activity significantly decreased. Our data is novel in that it shows COVID-19 severity is associated with both neutrophil elastase neutralization, together with complement activation.

## Introduction

Coronavirus disease 2019 (COVID-19) is associated with a strong innate immune response [1], mediated by neutrophils, lymphocytes, and the complement system. Severe COVID-19 involves the acute replication of severe acute respiratory syndrome coronavirus 2 (SARS-CoV-2) that results in a strong innate inflammatory response associated with damage to the airways, hyperactivation of the complement system, and multiorgan failure [2–4]. This innate immune response is mediated by monocytes, macrophages and neutrophils and an elevation in serum chemokines.

Neutrophil elastase and granzyme B were previously identified as significantly elevated in severe COVID-19 [5]. Similarly, other studies have shown an upregulation of neutrophil degranulation proteins in SARS-CoV-2 positive nasal swabs [6] and that neutrophil serine proteases are key mediators in the innate and adaptive immune functions of immune cells [7] and inflammatory disease [8]. Dysregulated proteolysis is central to inflammatory disease and examining the anti-protease and complement responses could provide a better understanding of the disease process and aid the development of targeted therapies.

The anti-protease activities of serine protease inhibitors (serpins) have been identified as potential clinical biomarkers of COVID-19 severity [9]. Proteomic methods routinely use immunoassays and ligand binding assays to measure protein abundance, but these methods are designed to quantify total protein levels and not protein activity. Studying serpins poses a proteomic challenge as structural information is required to distinguish between active and inactive forms.

Quantifying protein activity would provide a more accurate measure to investigate the function of these factors in the innate immune response in COVID-19 [10, 11]. Mass spectrometry enables functional proteomics to inform on protein structure and functional status for select proteins. Additionally, mass spectrometry is a powerful proteomics tool because it requires small sample sizes, is quantitative, and is amenable to customizable sample preparation and enrichment techniques.

In this study, we aimed to examine differences in serum levels of neutrophil elastase and granzyme B proteases, and to examine the anti-protease activity of their associated serpins, alpha-1-antitrypsin (ATT) and alpha-1-antichymotrypsin (ACT) as well as complement activation activity of factor 3 (C3) and complement factor 3 a (C3a) in context of COVID-19 severity. We present a functional mass spectrometry approach that allows active protein to be distinguished from total protein for further investigation of these potential COVID-19 biomarkers.

## Materials and methods

### Study participants and clinical data

Patients were enrolled March-June 2000 after admission to our participating academic hospital, London Health Sciences Centre (London, Ontario). COVID-19 was first suspected based on standard hospital screening procedures, and then confirmed as part of standard hospital testing by detection of two SARS-CoV-2 viral genes using polymerase chain reaction (Dual target assay, specific target unique to SARS-Cov-2 along with a conserved region of the E-gene; Roche cobas^®^ SARS-COV-2 Test). Patient baseline characteristics were recorded on admission and included age, sex, comorbidities, medications, hematologic labs, creatinine, arterial partial pressure to inspired oxygen (P/F) ratio, and chest x-ray findings. We calculated Multiple Organ Dysfunction Score (MODS) [12] and Sequential Organ Failure Assessment (SOFA) [13] score to illustrate their illness severity. We also recorded clinical interventions received during the observation period including use of antibiotics, anti-viral agents, systemic corticosteroids, vasoactive medications, venous thromboembolism (VTE) prophylaxis, anti-platelet or anti-coagulation treatment, renal replacement therapy, high flow oxygen therapy, and mechanical ventilation (invasive and non-invasive). For comparison to critically ill patients, we also included an age- and sex-matched non-critically ill COVID-19 patient cohort who were admitted to the hospital respiratory ward with moderate disease. All COVID-19 patients were non-vaccinated for SARS-CoV-2. Final participant groups contained were constructed by age- and sex-matching patient cohorts with healthy controls without disease, acute illness, or prescription medications (collected prior to the SARS-CoV-2 pandemic). Each of the three groups consisted of 15 participants.

### Blood draws

Standard operating procedures were used to ensure all samples were treated rapidly and equally (available at^1^). The first blood sample was obtained within 48 h of hospital admission via indwelling catheters using vacuum serum separator tubes and placed immediately on ice. For ICU patients, additional blood samples were drawn at 4, 7, and 10 days. If venipuncture was required, research blood draws were coordinated with a clinically indicated blood draw. In keeping with accepted research phlebotomy protocols for adult patients, blood draws did not exceed maximal volumes. Once transferred to a negative pressure hood, blood was centrifuged and sera isolated, aliquoted at 250 µL and frozen at −80°C. All samples remained frozen until use and freeze/thaw cycles were avoided.

### Protease measurements

The levels of serum neutrophil elastase and granzyme B, were determined using multiplexed biomarker immunoassay kits according to manufacturers’ instructions (MilliporeSigma, 400 Summit Drive, Burlington, MA), as we have previously described [5]. Analytes were measured using a Bio-PlexTM 200 Suspension Array system (Bio-Rad Laboratories, Hercules, CA), which used Luminex xMAPTM fluorescent bead-based technology (Luminex Corp, Austin, TX). Bioanalyte concentrations were calculated from standard curves using five-parameter logistic regression in Bio-Plex Manager 6.1 software. Serum concentrations are reported as pg/mL.

### Albumin and IgG depletion

A 60 mg aliquot of AlbuSorb™ PLUS beads (Biotech Support Group, Monmouth Junction NJ) was conditioned with 400 µL of Binding buffer (BB1), then the liquid was removed through a spin-filter by centrifugation for 2 min at 1000 × g, and repeated. A 25 µL aliquot of serum was diluted with 250 µL BB1 buffer, and clarified by passing through another spin-filter at 9,000 × g for 1 min. The clarified serum was loaded onto the conditioned AlbuSorb™ PLUS beads and incubated on a rotator at room temperature for 15 min then centrifuged for 4 min at 9,000 × g to collect the filtrate for further processing.

### Reduction and digestion

After depletion, an equal volume of serum filtrate ( ~20 µg) was loaded onto SDS-PAGE as gel plug, and in-gel digested with a standard protocol; proteins in the gel bands were reduced with 10 mM dithiothreitol (DTT) for 30 min at 60°C, alkylated with 20 mM iodoacetamide for 45 min at room temperature in the dark, and digested overnight with 0.4 µg of trypsin (Pierce MS Grade (ThermoFisher) at 37°C. Peptides were extracted twice with 5% formic acid, 60% acetonitrile and dried under vacuum.

### Target peptide selection

The target peptides were selected to report specific regions of the protein(s) that infer functionality (Table 1). For Complement C3, activation requires the proteolytic cleavage of the region C3a from the rest of the protein, generating Activated C3b. Two peptides were chosen from sequences of the C3 beta chain region – part of both the intact C3 and the Activated C3b subform (the C3 beta chain signal value was the average of the two peptides). One peptide was chosen from C3a sequence: the region proteolytically cleaved upon C3 Convertase activation [14]. As this method only considers observations at the protein level, less C3a relative to C3 beta would be indicative of proportionately more activated C3b subforms. For both Serpins (AAT and ACT), tryptic peptides which span the Reactive Center Loop (RCL) region were chosen, as these regions represent the proteins’ potential function as an inhibitor, any loss of which would negate inhibitory potential [14]. Taken together, the peptides selected from these regions thus can serve as surrogates for reporting functional activity.

**TABLE 1:** PRM target peptides for LC-MS/MS.

Protein	Target peptide sequence
Complement C3a region	FISLGEAC [+57]K
Complement C3 beta chain region	FVTVQATFGTQVVEK
Complement C3 beta chain region	IPIEDGSGEVVLSR
Alpha-1-Antitrypsin RCL Spanning Intact Region	GTEAAGAMFLEAIPMSIPPEVK
Alpha-1-Antichymotrypsin RCL Spanning Intact Region	ITLLSALVETR

### LC-MS/MS

0.2 µg of system-independent retention time (iRT) peptides (Biognosys) were added to each digested sample and 1% of the sample was analyzed by LC-MS/MS using a Dionex RSLC nano system coupled to an Orbitrap Eclipse Tribrid mass spectrometer (ThermoFisher). The peptides were loaded onto a fused silica trap column (Acclaim PepMap 100, 75 µm × 2 cm, ThermoFisher). After washing for 5 min at 5 μL/min with 0.1% trifluoroacetic acid (TFA), the trap column was brought in-line with an analytical column (Nanoease MZ peptide BEH C18, 130A, 1.7 µm, 75 µm × 250 mm, Waters) for LC-MS/MS. Peptides were fractionated at 300 nL/min using a segmented linear gradient 4–15% B in 5 min (where A: 0.2% formic acid, and B: 0.16% formic acid, 80% acetonitrile), 15–50% B in 50 min, and 50–90% B in 15 min. Parallel Reaction Monitoring (PRM) method template was used to target selected ions on the target list throughout the run (10–60 min). The target list is shown in Table 1. The isolation width was set at 1.6 Da. Automatic Gain Control (AGC) was set at 2E5 and max ion time set at 150 ms. Ions were fragmented by Higher Energy Collision Dissociation (HCD) using 30% relative collision energy and scanned in the Orbitrap with a resolution of 30,000 m/z. The data was analyzed using Skyline-daily (beta) [15] with a spectral library generated previously. Only peptides with dotp > 0.8 were accepted. All fragmented ions from the mass spectrum that corresponded to the target peptides, generate an accumulated spectral intensity. Relative spectral intensity data is normalized to the average value of the iRT peptide ions for each individual run. Complement C3 beta was reported as an average value of the two C3 beta peptides.

### Population statistics

Medians (IQRs) and frequencies (%) were used to report patient baseline characteristics for continuous and categorical variables, respectively; continuous variables were compared using Mann-Whitney U tests (and Kruskal-Wallis tests, as appropriate), and categorical variables were compared using Fisher’s exact chi-square tests. p-values <0.05 (*) were considered statistically significant. All analyses were conducted using GraphPad Prism version 9.2.0 for Windows (GraphPad Software, California, United States) and SPSS version 27 (IBM Corp., Armonk, NY, United States).

## Results

### Participant demographics and clinical variables

We investigated a total of 45 age- and sex-matched subjects, including 15 severe COVID-19 ICU patients, 15 mild COVID-19 ward patients and 15 healthy subjects. The mean age and sex distribution were similar between groups. Subject demographics and clinical variables are presented in Table 2. COVID-19 patients were likely infected with wild-type SARS-CoV-2 (B.1 strain) given the dates of recruitment and sample collection.

**TABLE 2:** Subject demographics and clinical variables.

Clinical variable	Healthy subjects	Mild COVID-19	Severe COVID-19
Age in years, median (IQR)	61 (46–66)	59 (46–68)	60 (47–67)
Sex, female: male	7:8	7:8	7:8
Comorbidities, n (%)			
Diabetes Hypertension Coronary Artery Disease Heart Failure Chronic Kidney Disease Cancer COPD		3 (20.0) 6 (40.0) 0 (0) 0 (0) 1 (6.6) 1 (6.6) 0 (0)	3 (20.0) 5 (33.3) 0 (0) 0 (0) 0 (0) 1 (6.6) 0 (0)
MODS, median (IQR)		-	4.5 (4.0–5.0)
SOFA, median (IQR)		-	6.0 (2.0–7.0)
Presenting Symptoms, n (%)			
Fever Cough Anosmia/ageusia Pharyngitis Headache Myalgias Dyspnea Chest Pain Nausea/vomiting/diarrhea		11 (73.3) 12 (80.0) 3 (20.0) 3 (20.0) 2 (13.3) 9 (60.0) 13 (86.6) 0 (0) 6 (40.0)	- - - - - - - - -
Laboratory, median (IQR) Hemoglobin White Blood Cell Count Neutrophils Lymphocytes Platelets Creatinine INR Lactate CRP D-Dimer Albumin		137.0 (117.0–142.0) 7.5 (4.7–10.4) 6.6 (3.5–8.4) 1.0 (0.5–1.2) 236.0 (187.0–277.0) 70.0 (54.0–92.0) 1.0 (1.0–1.1) 1.5 (1.3–2.2) 90.6 (68.1–112.1) 616.0 (441.0–3,367.0) 34.0 (31.5–36.5)	121.0 (104.0–136.0) 8.2 (6.9–11.5) 7.3 (5.5–9.7) 0.7 (0.4–1.0) 210.0 (186.0–210.0) 81.0 (56.0–184.0) 1.2 (1.1–1.3) 1.2 (1.0–1.9) 133.9 (71.1–240.5) 3,964.0 (1224.0–14725.0) 30.0 (26.0–32.0)
Pulmonary Abnormalities, n (%)			
Bilateral Infiltrates Unilateral Infiltrates Normal		14 (93.3) 0 (0) 1 (6.6)	15 (100) 0 (0) 0 (0)
P/F Ratio, median (IQR)		-	93.0 (66.0–131.0)
Interventions, n (%)			
Antibiotics Steroids Remdesivir Tocilizumab Vasoactive medications High Flow Nasal Cannula Non-invasive MV Invasive MV Renal Replacement Therapy		14 (93.3) 15 (100) 4 (26.6) 1 (6.6) 2 (13.3) 7 (46.6) 1 (6.6) 2 (13.3) 0 (0)	15 (100) 10 (66.6) 0 (0) 0 (0) 14 (93.3) 10 (66.6) 4 (26.6) 14 (93.3) 3 (20.0)
ICU Days, median (IQR)		-	17.0 (10.0–26.0)
Hospital Days, median (IQR)		8.0 (6.0–16.0)	-
Outcome, n (%)			
Alive Dead		15 (100) 0 (0)	8 (53.3) 7 (46.6)

### Serine protease measurements

**FIGURE 1:**
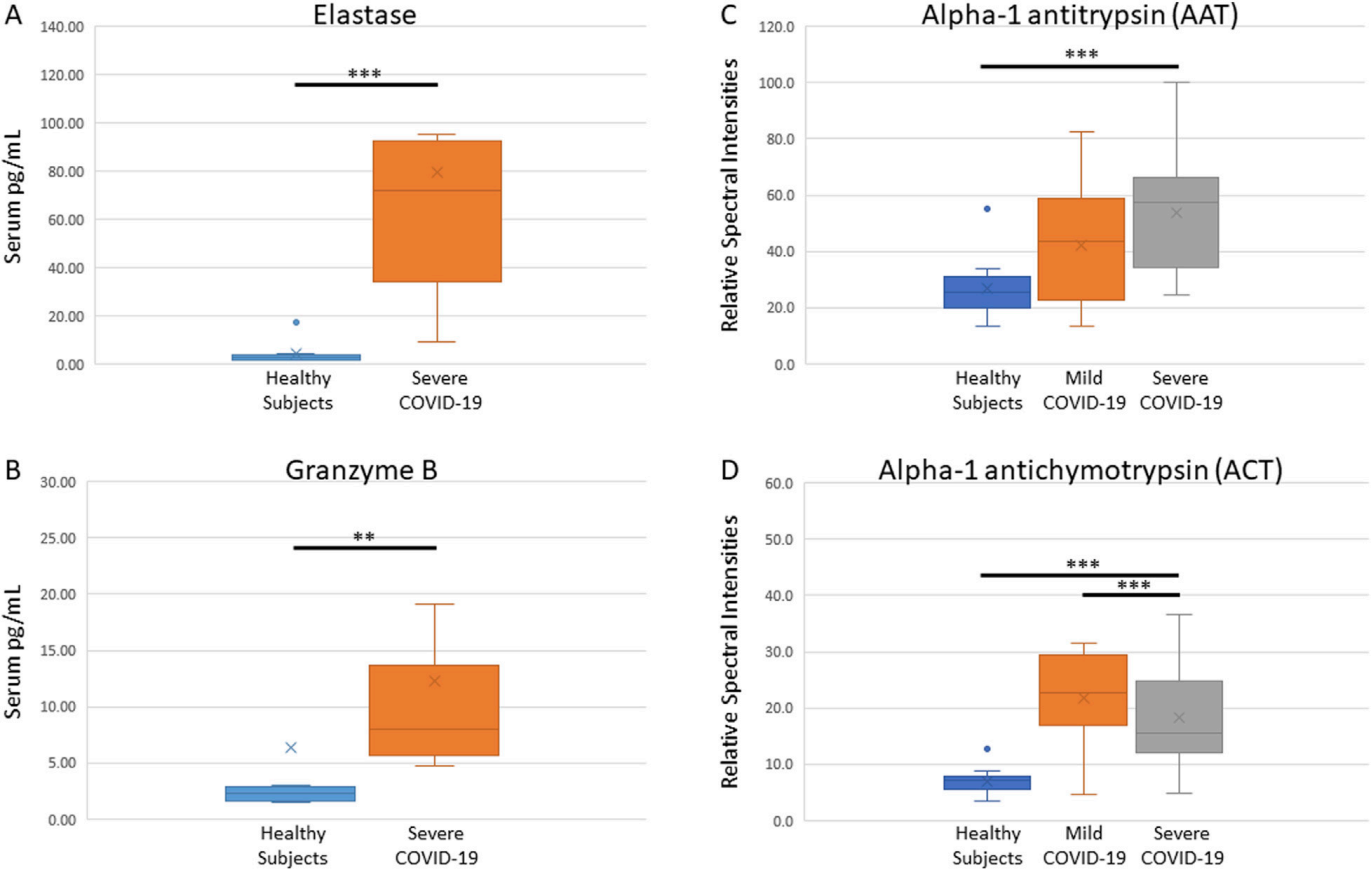
Measurements of serine proteases and functional analyses of their anti-proteases. Immunoassays show elevated levels of neutrophil elastase **(A)** and granzyme B **(B)**, and functional proteomic analysis indicate high activity of AAT **(C)** and ACT **(D)** anti-proteases, in serum of COVID-19 infected patients within 48 h of hospital admission.

We observed increased levels of neutrophil elastase and granzyme B in patients hospitalized for severe COVID-19. Immunoassay quantification of these proteases in serum indicate that within 48 h of admission, severe COVID-19 patients had significantly higher levels of neutrophil elastase (median 71.8 pg/mL versus 2.8 pg/mL; p > 0.001) (Figure 1A) and granzyme B (median 8.0 pg/mL versus 2.3 pg/mL; p = 0.006) (Figure 1B).

### Anti-protease activity

Functional mass spectrometry was used to quantify the level of active serpins AAT and ACT in serum. We observed that within 48 h of hospital admission, anti-protease activity in COVID-19 patient serum is significantly increased. AAT activity was highest in severe COVID-19 patients (median 57.3, p < 0.001; Figure 1C) and ACT activity (median 22.6, p < 0.001; Figure 1D) was significantly higher in mild compared to severe cases. These findings indicate that compared to healthy subjects, AAT may be correlated with COVID-19 disease severity. Interestingly, ACT activity was elevated in mild COVID-19 but decreased in severe COVID-19 serum (median 15.6, p < 0.001). Although ACT is significantly elevated in serum of both the mild and severe COVID-19 population, ACT may be relatively depleted in serum with a greater protease load. Neither AAT nor ACT changed significantly over 10 days of ICU admission (AAT, p = 0.082; ACT p = 0.059).

### Complement activation

**FIGURE 2:**
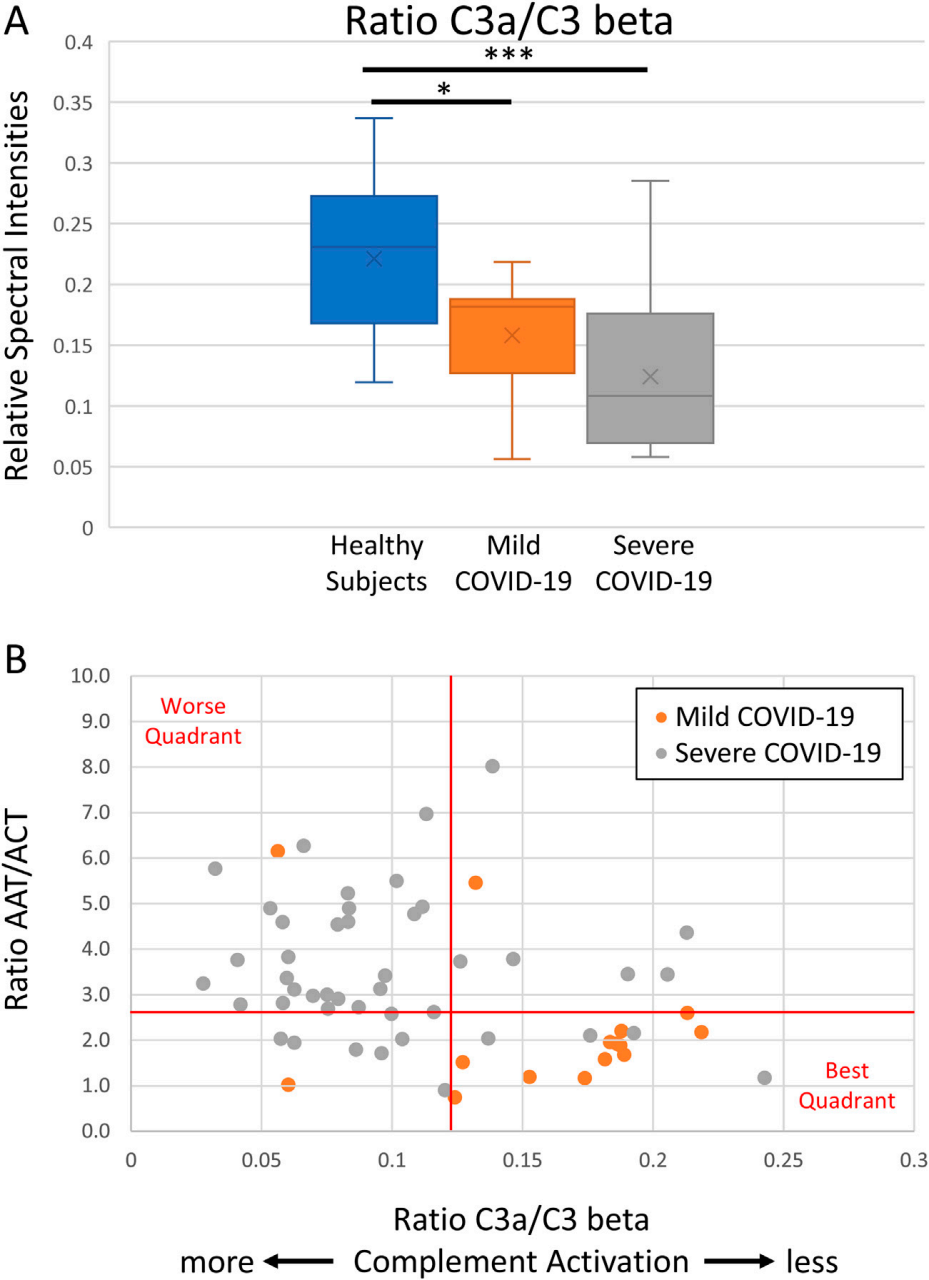
Functional analyses of complement activity and its role in COVID-19 severity. Functional proteomic analysis of serum complement activity indicates a decrease in C3a/C3 beta ratio **(A)**, which is a metric of relative C3 activation, and a comparison of anti-protease activity to complement activation indicates milder COVID-19 cases cluster toward low anti-protease activity ratio (AAT/ACT; the ratio of the RCL tryptic peptides) and less complement activation (C3a/C3 beta) **(B)**. The graphical line thresholds were chosen arbitrarily as an indicator for quadrant stratification. Severe COVID-19 data points were calculated from all ICU days.

We observed that serum Complement activation was associated with COVID-19 severity within 48 h of hospital admission, as inversely measured by the C3a/C3beta ratio (median 0.12, p < 0.001) (Figure 2A). The C3a/C3beta ratio did not change significantly over 10 days of ICU admission (p = 0.447; data not shown).

### Consequences of anti-protease activity versus complement activation

We compared anti-protease activity ratio (AAT/ACT) to complement activation (C3a/C3 beta) in both mild and severe COVID-19 cases. Our results indicate that milder COVID-19 cases cluster towards the lower right quadrant and associated with low anti-protease activity ratio and low complement activation (Figure 2B).

## Discussion

In this study, we investigated two serine proteases, previously identified as elevated in the serum of COVID-19 ICU patients [5], to assess their functional activity using mass spectrometry. We compared healthy controls to COVID-19 patients with mild or severe disease. Our results showed increased anti-protease activity with disease severity and decreased complement activity in COVID-19 patients.

Participants were classified as mild or severe based on PCR results and symptom severity, all infected with wild-type SARS-CoV-2. Blood samples were collected within 48 h of hospital admission. While all mild patients survived, 47% of severe patients died. Healthy controls were obtained prior to the pandemic.

Immunoassays showed significantly higher levels of neutrophil elastase and granzyme B in severe COVID-19 patients within 48 h of admission, compared to healthy controls. These proteases are crucial for immune regulation, but excessive activity can cause tissue damage, cytotoxicity, and inflammation [7, 16]. Protease-induced degradation of the extracellular matrix also damages blood vessels, increasing vascular permeability and causing hypotension, often necessitating vasoactive support. The elevated neutrophil elastase seen in COVID-19 may contribute to NETosis, where neutrophils release extracellular traps, further driving inflammation and tissue damage [17].

AAT is the primary inhibitor of neutrophil elastase, a serine protease released by neutrophils, monocytes, macrophages, and mast cells during immune responses to kill pathogens [18]. Neutrophil elastase activates receptors to increase chemokines and proinflammatory cytokines, promoting leukocyte recruitment [19, 20]. Elevated elastase contributes to cell death, inflammation, pulmonary vascular permeability, and injury [21] and is linked to conditions like chronic obstructive pulmonary disease [22], acute lung injury [23], and acute respiratory distress syndrome [24]. The D614G mutation in the SARS-CoV-2 spike protein created a cleavage site for elastase, enhancing viral spread, particularly in AAT-deficient individuals [25].

Granzyme B, a serine protease in cytotoxic T and natural killer cells, shares structural similarities with chymotrypsin and may be inhibited by AAT [16]. These cells release granzyme B via exocytosis to induce rapid cell death in infected cells [26]. Granzyme B can also function extracellularly, promoting proinflammatory cytokine responses and tissue remodeling that can lead to tissue destruction [27–30]. As a pro-apoptotic factor, granzyme B activates cytokines like Interleukin-18, which amplifies the innate immune response [31].

To evaluate the therapeutic potential of granzyme B and neutrophil elastase, we assessed their anti-protease and complement activity in COVID-19 severity using functional mass spectrometry. Serpin inhibition involves a 1:1 complex formation that irreversibly inactivates both the serpin and protease [32, 33]. The RCL of the serpin targets specific proteases, mimicking their substrate sequence, leading to cleavage, a conformational change, and the permanent deactivation of both proteins [34].

Immunoassays cannot differentiate between active and inactivated serpins, but functional mass spectrometry can detect peptide sequences and assess the state of the RCL region [35]. This study identified distinct peptide sequences in the RCL regions of AAT and ACT, associated with elastase and granzyme B, respectively. Intact peptide sequences in the RCL represent active anti-protease activity. Protein depletion beads were used to remove albumin and IgG from serum, enriching lower-abundance proteins for proteomic analysis.

Our results show that AAT significantly increases within 48 h of hospital admission in COVID-19 patients, correlating with disease severity and elevated neutrophil elastase levels. This aligns with previous studies showing increased serpins, including AAT, in COVID-19 sera, particularly in patients with high Interleukin-6 levels [36], and evidence suggesting that AAT deficiency increases the risk of severe COVID-19 [37].

The anti-protease and anti-inflammatory activity of AAT [38] may help mitigate COVID-19 and the hyperactive immune response. AAT inhibits SARS-CoV-2 entry by inactivating TMPRSS2, a serine protease that primes the spike protein for cell surface fusion [39]. AAT also protects against cytotoxicity by inhibiting inflammatory proteases, particularly neutrophil elastase [36]. Our results show that within 48 h of hospital admission, ACT activity increased with COVID-19 infection, peaking in mild cases. In severe COVID-19, ACT activity decreased, likely due to higher granzyme B and other proteases, leading to greater proteolysis that may inactivate ACT and compromise its regulatory function.

ACT is a serpin that inhibits chymotrypsin-like proteases, including neutrophil cathepsin G and mast cell chymases [40, 41]. Its concentration increases during acute inflammation and may protect tissues, such as the lower respiratory tract and brain, from proteolytic damage [42]. In cell culture, ACT-cathepsin G complexes signal acute phase protein synthesis and increase interleukin-6 production by fibroblasts [43]. Since ACT irreversibly forms complexes with cathepsin G, further research is needed to determine whether ACT activity protects against or contributes to severe COVID-19.

ACT is also a substrate for neutrophil elastase and other proteases [44]. In these cases, the protease cleaves ACT without forming an irreversible complex, rendering ACT inactive. Cleaved ACT products become potent chemoattractants at nanomolar concentrations [45], with slower serum clearance than ACT-cathepsin G complexes, suggesting prolonged neutrophil chemotaxis and potential interleukin-6 (IL-6) stimulation. Elevated IL-6 is linked to acute COVID-19 symptoms, including complement cascade upregulation, and current COVID-19 treatment regimens include the use of the IL-6 specific monoclonal antibody Tocilizumab [36].

C3a, a small complement fragment, is an anaphylatoxin that regulates inflammation with both pro- and anti-inflammatory effects [46]. It triggers chemokine release through mast cell degranulation and increased vascular permeability, but also helps regulate B-cell immune responses. C3 cleavage to form C3a is a key step in all complement pathways, necessary for C3 convertase to generate the membrane attack complex (C5b-9), which induces cell lysis and death. At sublytic levels, C5b-9 regulates tissue homeostasis by influencing cell cycle, proliferation, and differentiation [47]. C3 can also be cleaved by various serine proteases, including thrombin and cathepsin, at sites of inflammation [48]. As an anaphylatoxin, C3a increases vascular permeability and causes vasodilation, which can result in hypotension. The latter requires vasoactive support.

Multiple studies suggest that complement activation plays a key role in COVID-19 pathology [49], with C3 split products (e.g., C3a) often monitored by immunoassay [50, 51]. Using functional mass spectrometry, we distinguished cleaved C3b sequences from unprocessed C3 and examined the C3a/C3 beta; ratio as a relative metric for C3b activation, with lower ratios indicating greater activation. A limitation of this method is the use of a small 9-amino acid C3a peptide (FISLGEACK) compared to the full 77-amino acid C3a. Future studies are needed to confirm whether this peptide is sufficient for monitoring C3 subforms and to compare results with immunoassay measurements.

Our findings align with previous studies, showing an inverse relationship between the C3a/C3 beta ratio and COVID-19 severity. This suggests that anti-complement therapy may benefit severe COVID-19 patients. The mass spectrometry approach allows for efficient examination of functional characteristics, such as post-translational modifications and genetic variations, which are challenging for immunoassays. By comparing anti-protease activity (AAT/ACT ratio) to complement activation (C3a/C3 beta ratio), mild COVID-19 cases clustered in the lower right quadrant, indicating low anti-protease activity and minimal complement activation. These clusters may provide insights into disease severity and help predict disease progression and optimal interventions.

The COVID-19 pandemic and long COVID highlight the need for therapeutics that target viral infection and modulate immune responses. Serine proteases and serpins are promising drug targets for immunomodulation [52]. Future studies on the balance between protease and anti-protease activities, particularly AAT/ACT ratios, may reveal insights into enzyme regulation, complement activation, and clinical outcomes. The AAT/ACT ratio could serve as both a biomarker for COVID-19 severity and a potential therapeutic target.

## Data Availability

The raw data supporting the conclusions of this article will be made available by the authors, without undue reservation.

## Footnotes

1
https://translationalresearchcentre.com/
